# *In vitro* growth-inhibitory effects of *Portulaca oleracea* L. formulation on intestinal pathogens

**DOI:** 10.1099/acmi.0.000208

**Published:** 2021-02-24

**Authors:** Sae Okuda, Takeaki Wajima, Tetsuya Yamada, Hidemasa Nakaminami, Hideaki Ikoshi, Norihisa Noguchi

**Affiliations:** ^1^​ Department of Microbiology, School of Pharmacy, Tokyo University of Pharmacy and Life Sciences, Tokyo, Japan; ^2^​ Department of Traditional Chinese Medicine, School of Pharmacy, Tokyo University of Pharmacy and Life Sciences, Tokyo, Japan

**Keywords:** *Portulaca oleracea *L., *Shigella dysenteriae*, *Vibrio cholerae*, Cholera, Dysentery

## Abstract

**Introduction:**

Empirical evidence suggests that *Portulaca oleracea* L. treats enteric infections, including dysentery, cholera, and acute infectious gastroenteritis.

**Aim:**

The aim of this study is to clarify the growth-inhibitory effects of *Portulaca oleracea* L. extract against 56 strains of intestinal pathogens.

**Methodology:**

‘Gogyo-so-cha (GSC)’ was used as the *P. oleracea* L. formulation. A growth curve analysis was used to measure the growth-inhibitory effects of GSC, and Shiga toxin induction was measured using the latex agglutination test.

**Results:**

GSC demonstrated strong bactericidal effects against *Shigella dysenteriae* and *Vibrio cholerae* strains from various isolates. GSC demonstrated weak or no bactericidal effects against intestinal commensal bacteria, including *Enterococcus* spp. and *Escherichia coli *. GSC did not induce *Shigella* toxins.

**Conclusion:**

GSC significantly inhibited the growth of intestinal pathogens, including *S. dysenteriae* and *V. cholerae *, without adversely affecting the intestinal flora, supporting the usage of GSC in traditional Chinese medicine. Taken together, GSC would be of immense value in the developing world, where diarrhoeal infectious diseases continue to pose a major health risk.

## Introduction

*Portulaca oleracea* L. is a perennial plant that is ubiquitously distributed from the tropical zone to the temperate zone and is marketed as a health food in Japan under the name Gogyo-so-cha. In traditional Chinese medicine, *P. oleracea* extract has been used empirically for the treatment of infectious diseases, particularly intestinal infections, including dysentery, cholera, and acute infectious gastroenteritis [1].

Dysentery and cholera are rare in developed countries owing to high standards of sanitation and education regarding hygiene but persist as serious health problems in developing countries, especially in Southeast Asia [2]. Epidemiological studies demonstrated that the number of dysentery patients exceeded 600000 in Bangladesh, China, Pakistan, Indonesia, Vietnam, and Thailand, and most of these patients were children under 5 years of age [2]. The World Health Organization (WHO) estimated the annual incidence of cholera to be between 1.3 million and 4 million, with there being between 2100 and 143000 deaths per year [3].

Dysentery is usually treated with antimicrobial agents. Although these agents are effective, they can induce the release of intracellular toxins and toxin gene expression [4, 5]. Antimicrobial therapy is not suitable for cholera, and oral replacement of fluids is the treatment of choice [6]. An effective therapeutic agent that does not cause toxin release is required; however, the development of novel antimicrobial agents is on the decline [7].

Randomised controlled trials reported success regarding the use of probiotics for cholera prophylaxis [8]. There are also reports indicating that bacteriostatic drugs are effective for the treatment of intestinal infections [9]. These results suggest that agents with milder actions may be more efficacious than standard treatments for enteric infections.

We thus investigated the antimicrobial efficacy of the *P. oleracea* formulation, Gogyo-so-cha (GSC), against various intestinal pathogens to determine its usefulness as a therapeutic agent.

## Methods

### Bacterial strains, culture conditions, and chemicals

A total of 56 bacterial strains from 20 species were investigated that included control strains and various isolates (Table 1). All strains, except anaerobic bacteria, *Enterococcus* spp., and *Candida albicans*, were cultured at 35 °C in Mueller–Hinton broth and agar (Oxoid, Hampshire, UK). For anaerobic bacteria, modified Gifu Anaerobic Medium (GAM) broth and agar (Nissui Pharmaceutical, Tokyo, Japan) were used. For *Enterococcus* spp. and *Candida albicans*, brain heart infusion (BHI) broth and agar (Oxoid) were used. Anaerobic bacteria were cultured under the recommended conditions. Antimicrobial susceptibility and inhibitory effects against these strains were confirmed by previous study [10, 11].

**Table 1.** Bacterial and fungal strains used in this study

Species	Strain	Species	Strain
Gram-positive			
* Staphylococcus aureus *	N315*	* Bacillus subtilis *	ATCC6633*
* Enterococcus faecalis *	JCM5803*	* Enterococcus faecium *	JCM5804*
* Clostridioides difficile *	JCM11019*		
Gram-negative			
* Escherichia coli *	ATCC25922*	* Enterobacter cloacae *	JCM1232*
	Str 3860 (ESBL producer)†		Str 3929 (MBL producer)**
	Str 3863 (ESBL producer)†		Str 4087 (MBL producer)**
	Str 3865 (ESBL producer)†	* Citrobacter freundii *	JCM1657*
	E96164 (O157: H7)		Str 4000 (MBL producer)**
	EDL933 (O157: H7)		Str 4086 (MBL producer)**
	4266 (LT・ST producer)	* Bacteroides fragilis *	JCM1296*
	86–24 (O157: H7)	* Vibrio cholerae *	RIMD2203098 (O1, Ogawa)*
* Klebsiella pneumoniae *	ATCC13883*		RIMD2214034 (non-O1)
	Str 1449 (MBL producer)†		RIMD2203088 (O1, Inaba)
	Str 3333 (MBL producer)†		569B (O1, classical)
	Str 3740 (ESBL producer)†		SG24 (O139)
	Str 3750 (ESBL producer)†		N16961 (O1, Eltor)
	Str 3750 (ESBL producer)†	* Shigella dysenteriae *	4379–90*
	Str 3859 (ESBL producer)†		GTC00786T
* Klebsiella oxytoca *	JCM1665*		GTC01930
	Str 3789 (ESBL producer)†		NT4907
	Str 3790 (ESBL producer)†		BCH518
	Str 3811 (ESBL producer)†	* Shigella flexneri *	GTC00780T*
* Proteus mirabilis *	JCM1669*		IID642
	Str 3232		GTC01924
	Str 3830 (ESBL producer)†	* Shigella boydii *	GTC0079T*
	Str 3831 (ESBL producer)†		GTC01914
	Str 3858 (ESBL producer)†	* Shigella sonnei *	GTC00781T*
* Serratia marcescens *	JCM1239*		GTC01911
		* Salmonella* Enteritidis	PTI-93–417*
Fungi			
*Candida albicans*	ATCC10231*		

*These strains are either type strains or susceprible control strains.

†Clinical isolates from Tokyo medical university Hachioji medical center.

ESBL, Extended spectrum β-lactamase; MBL, metallo-β-lactamase; LT, heat-labile enterotoxin; ST, heat-stable enterotoxin.

GSC (Lot, 2020.1/FCA IK; Iskra Co. Ltd., Tokyo, Japan) was employed as the *P. oleracea* formulation and contained 3 g (dry weight) of condensed *P. oleracea* extract per gram and no other bioactive components.

### Evaluation of growth-inhibitory effect

The growth-inhibitory effect was measured as previously described [11]. Tested strains were suspended in the appropriate media, as described above, and cultured overnight at 35 °C with shaking. The bacterial concentration was adjusted to 10^3^ c.f.u. ml^−1^ with the same medium, and cultures were incubated at 35 °C with shaking in the presence or absence of GSC (20 mg ml^−1^). After 1-, 2-, 4-, and 6 h incubation periods, the cultures were diluted with PBS and spread onto appropriate agar media. On the following day, colony number was counted, and bacterial concentration (c.f.u. ml^−1^) was calculated. In cases where anaerobic bacteria and fungi were assessed, c.f.u. ml^−1^ was also calculated after 24 h of incubation. For bacteria that were inhibited by GSC, assays were performed at least three times on different days. The log reduction value (LRV) was calculated as described below:

LRV=log (c.f.u. in the absence of GSC / c.f.u. in the presence of GSC)

### Evaluation of the effect of GSC on Shiga toxin production

*Shigella dysenteriae* BCH 518 was cultured overnight and then diluted with fresh LB (1 : 100) and cultured with shaking for 6 h at 35 °C. GSC was then added at a concentration of 20 mg ml^−1^. For comparison, 5 mg ml^−1^ levofloxacin (L0193; Lot, 8NMZJ-TS; Tokyo Chemical Industry, Tokyo, Japan) and 5000 units ml^−1^ polymyxin B (167–11691; Lot, LAJ6280; Wako, Osaka, Japan) were also added to *S. dysenteriae* solutions as experimental controls. The mixtures were incubated for 30 min at 35 °C with shaking. The bacteria were removed by centrifuging at 4000 ***g*** for 20 min. Toxin level in the supernatant was quantified using a Shiga Bacterial Toxin Detection Kit VTEC-RPLA ‘Seiken’ (DENKA SEIKEN, Tokyo, Japan).

### Statistical analysis

Differences in growth-inhibitory effect and Shiga toxin production were evaluated in the presence and absence of GSC by Welch’s *t*-test. *P*<0.05 was considered significant.

## Results and discussion

### Growth-inhibitory effects of GSC against various bacteria and fungi

**Fig. 1.**
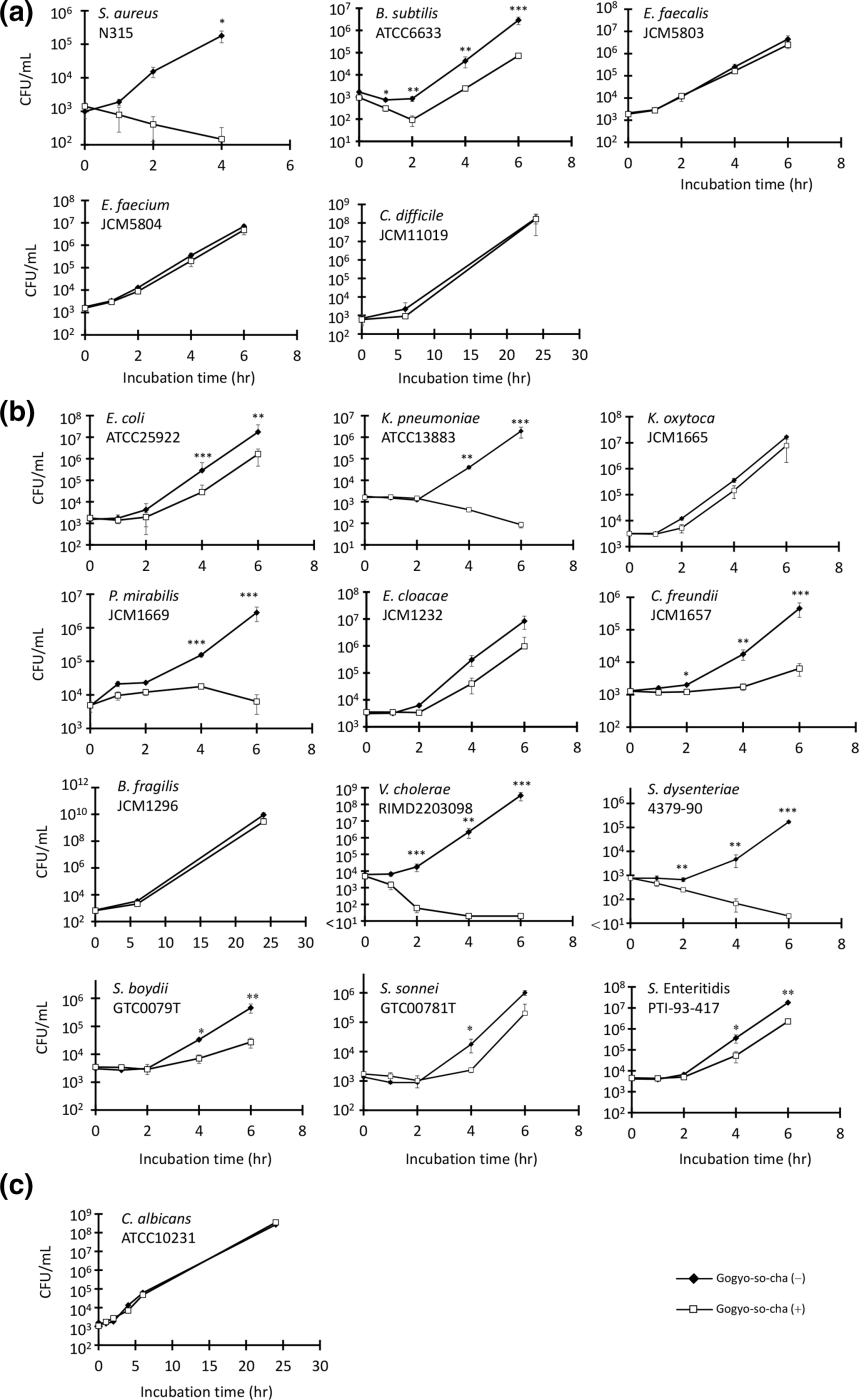
The growth-inhibitory effect of Gogyo-so-cha for the type or susceptibility test standard strains of various microorganisms (a), Gram-positive bacteria; (b), Gram-negative bacteria; (c), fungi. Data are presented as mean±SD and level of significance is indicated as *P<0.05, **P<0.01, ***P<0.001.

We measured growth-inhibitory effects of GSC against various bacteria and fungi using the methods that were confirmed by previous studies [10, 11] (Fig. 1). GSC significantly reduced the growth rates of *Bacillus subtilis *, *Escherichia coli *, *Citrobacter freundii *, *Shigella boydii *, *Shigella sonnei *, and *Salmonella* Enteritidis (*P*<0.01 for all) and inhibited the growth of *Staphylococcus aureus *, *Klebsiella pneumoniae *, *Proteus mirabilis *, *Vibrio cholerae *, and *Shigella dysenteriae *. GSC did not inhibit *Enterococcus faecalis *, *Enterococcus faecium *, *Clostridioides difficile *, *Klebsiella oxytoca *, *Serratia marcescens *, *Enterobacter cloacae *, *Bacteroides fragilis *, and *Candida albicans*. These data demonstrated GSC’s inhibitory effects against intestinal pathogens, supporting its use as an empirical therapy.

### Growth-inhibitory effects of GSC against intestinal pathogens

**Fig. 2.**
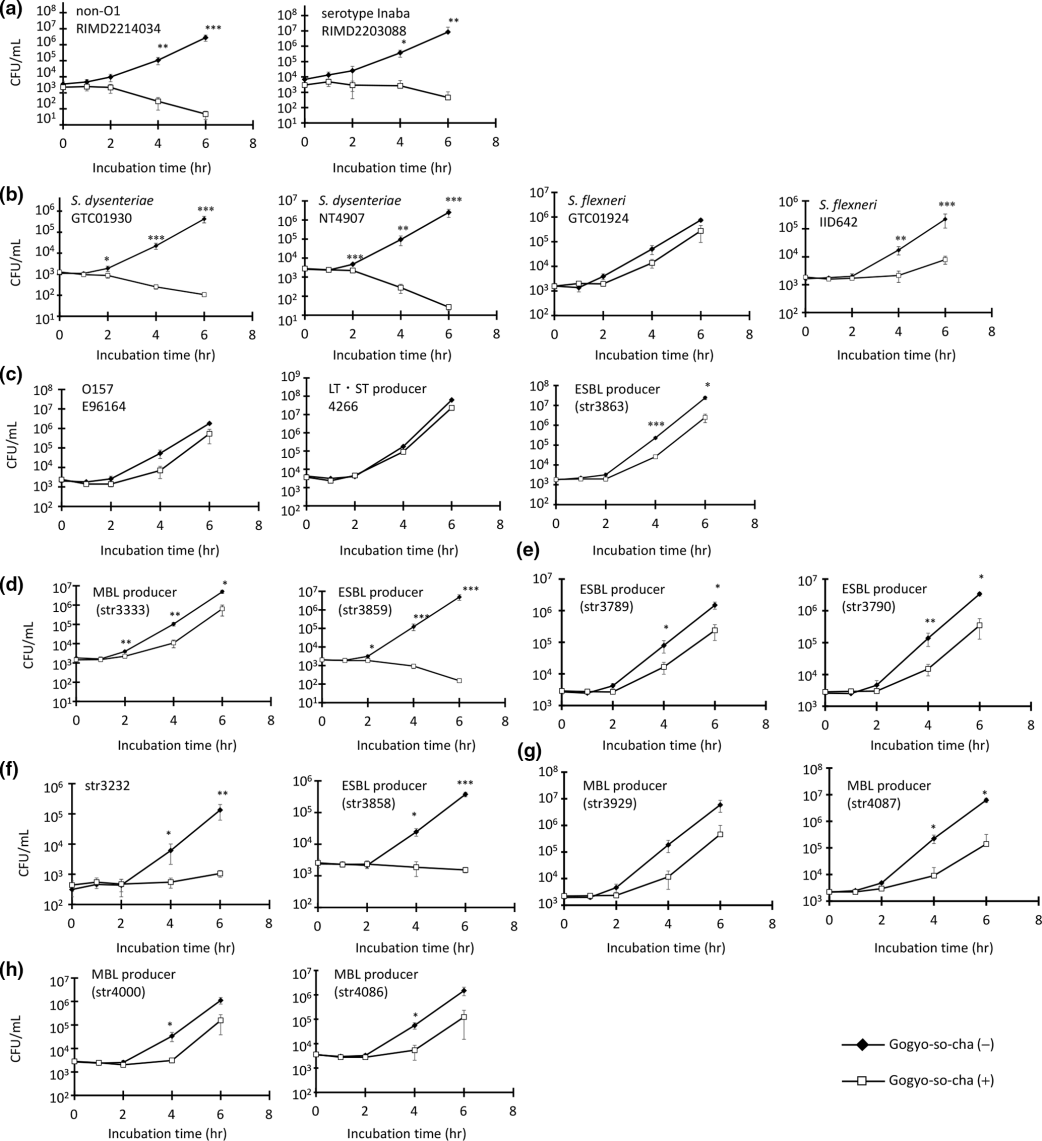
Inhibitory effect of Gogyo-so-cha on strains from clinical isolates and other strains of intestinal bacteria **(a), *V *. cholerae** *;* (b), *Shigella* spp.; (c) *E. coli *; (d), *K. pneumoniae *; (e), *K. oxytoca *; (f), *P. mirabilis *; (g), *E. cloacae *; (h), *C. freundii *. Data are presented as mean±SD and level of significance is indicated as *P<0.05, **P<0.01, ***P<0.001.

We used clinical isolates, including antimicrobial-resistant strains, to confirm the growth-inhibitory effects of GSC against intestinal pathogens (Fig. 2). The growth rates of all tested strains of *V. cholerae* (*n*=5) and *S. dysenteriae* (*n*=4) were inhibited significantly. GSC weakly suppressed the growth of *S. flexneri *. However, comparing the CFUs at 24 h after incubation, there was no significant changes regardless of the presence or absence of GSC (data not shown), suggested that GSC temporary delayed *S. flexneri *. These data suggested that the growth-inhibitory effects of GSC were different for *Shigella* spp. We investigated the effects of GSC against pathogenic *E. coli *, including enterotoxigenic (ETEC), enterohaemorrhagic *E. coli* (EHEC), and expanded spectrum β-lactase (ESBL)-producing *E. coli *. The growth of ESBL-producing clinical isolates was inhibited significantly by GSC, although no inhibitory effects against EHEC and ETEC were observed.

The growth rates of metallo β-lactamase (MBL)-producing and ESBL-producing *K. pneumoniae* clinical isolates (*n*=8) were reduced significantly by GSC. Additionally, the growth rates of *K. pneumoniae* and *P. mirabilis* clinical isolates, including ESBL producers (*n*=4) and a *C. freundii* clinical isolate (MBL producer), were also inhibited. There was no growth inhibition seen for *E. cloacae* clinical isolates.

The cause of variation in antimicrobial effects among species in the *Enterobacteriaceae* family remains unclear. *P. oleracea* contains many components, including flavonoids, alkaloids, fatty acids, terpenoids, and polysaccharides [12, 13]. Previously, purified portulacerebrosides, novel cerebroside compounds isolated from *Portulaca oleracea* L., were reported to exert antimicrobial activity against enterobacteria [14]. The interactions between the many compounds comprising GSC may account for variations in its antimicrobial effects.

Collectively, our data indicated that GSC has strong bactericidal growth-inhibitory activity against *S. dysenteriae* and *V. cholerae* strains from various clinical isolates. However, GSC showed no inhibitory effect (or weak or temporary effect) against commensal bacteria, including *E. faecalis *, *E. faecium *, *E. coli *, and *K. oxytoca *.

The pH value of Mueller–Hinton medium was 7.18 without GSC and 6.68 with GSC. Since pH significantly affects bacterial growth, we performed a growth-inhibition assay using low-pH (pH 6.68) Mueller–Hinton medium (data not shown). The growth-inhibitory effect on *E. coli *, *S. dysenteriae *, and *V. cholerae* in the pH 6.68 Mueller–Hinton medium remained unchanged, suggesting growth inhibition was attributable to GSC and not the decrease in pH caused by GSC addition to Mueller–Hinton medium.

### Comparison of bacterial number LRV

**Fig. 3.**
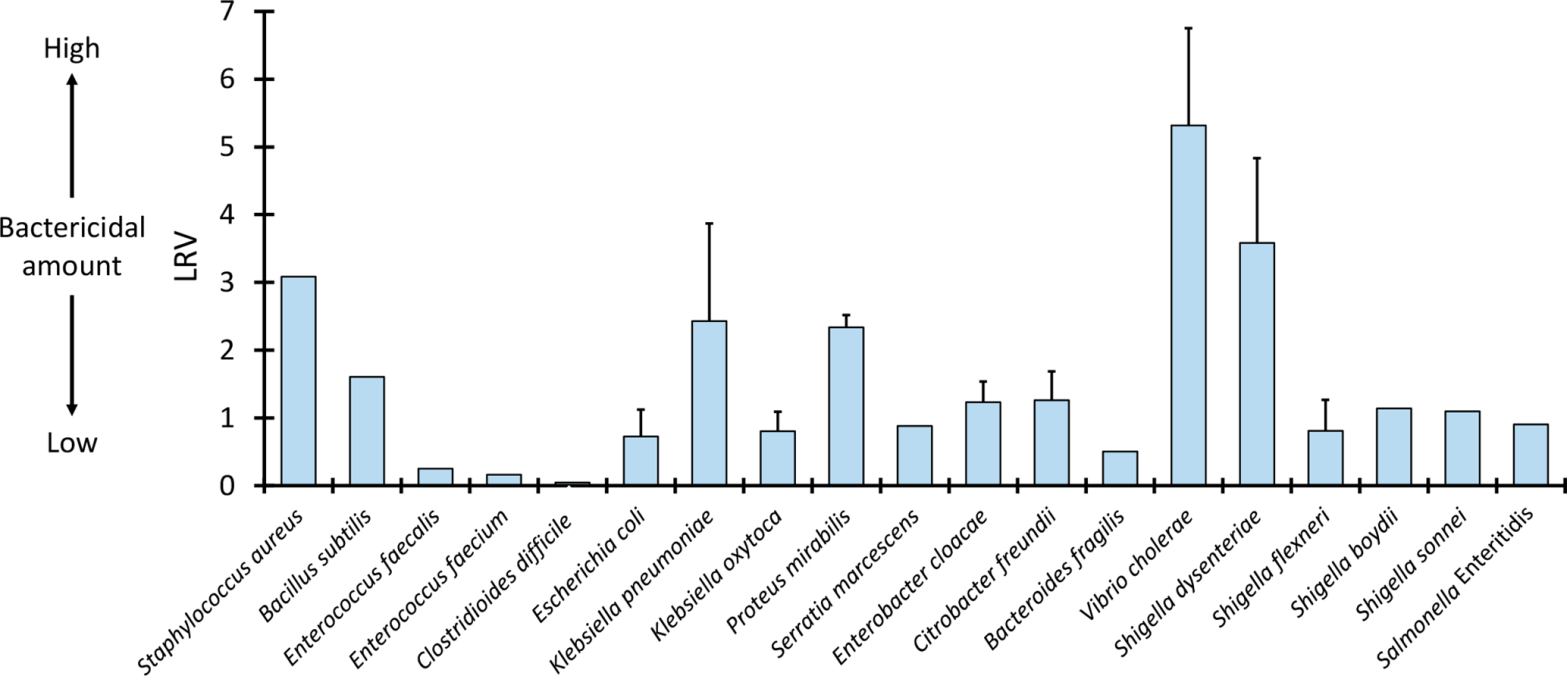
Log reduction value (LRV) at the end of culture Values are the mean of all strains of a species. Bars indicate+SD.

The LRV for each bacterial species was calculated at 6 and 24 h to evaluate the antimicrobial spectrum of GSC and compare bacterial numbers between species (Fig. 3). The LRVs for *V. cholerae* and *S. dysenteriae* were found to be greater than 3, showing the potent bactericidal action of GSC.

### Evaluation of toxin-inducing ability

**Fig. 4.**
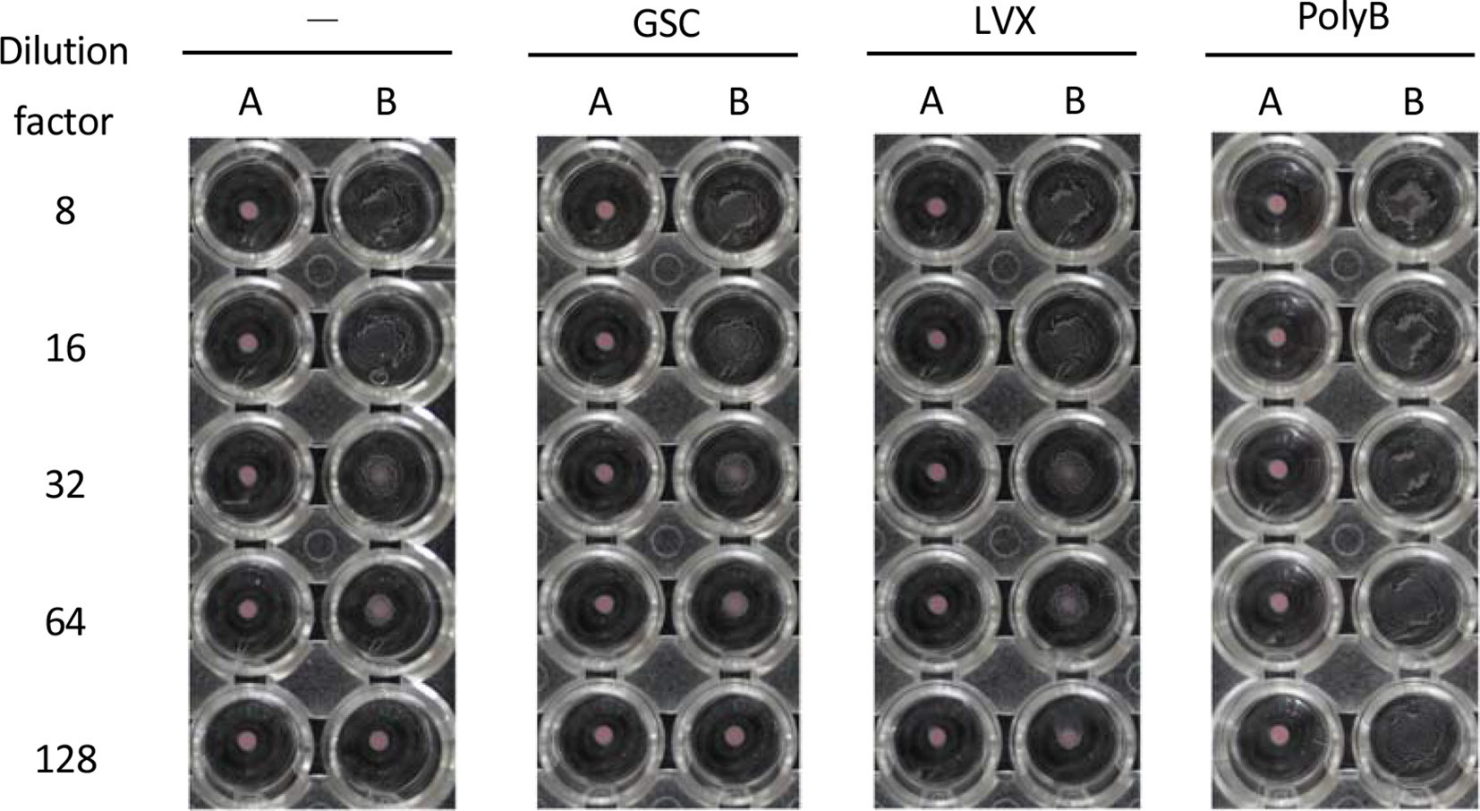
Productivity of Shiga toxin in the culture supernatant in the presence of Gogyo-so-cha. –, No drug; GSC, 20 mg ml^−1^ Gogyo-so-cha; LVX, 5 mg ml^−1^ levofloxacin; PolyB, 5000 unit ml^−1^ polymyxin B A, control latex; B, Sensitised latex VT1.

In general, a large portion of the Shiga toxin was found on the periplasm of *S. dysenteriae* rather than on its outer membrane. Some antimicrobial agents can cause bacteria to release intracellular toxins via cell lysis or induce toxin production via toxin-encoding bacteriophages, and this may negatively impact recovery [4, 15, 16]. To evaluate whether GSC induced bacteria to release or produce toxins, we compared the amount of toxin in the medium with polymyxin B, levofloxacin, or GSC (Fig. 4) When toxin production in the presence of polymyxin B and levofloxacin were assayed, toxins in the media were found to be present at more than 128 folds and 64 folds, respectively. However, when GSC was assayed, toxins were found to be present at up to 32 folds, and no difference between its activity and that of the control (no antimicrobial agent) was seen, indicating that GSC may not possess toxin-inducing ability.

## Conclusion

Our data indicated that GSC exerts strong antimicrobial effects against intestinal pathogens, including *S. dysenteriae* and *V. cholerae *. Moreover, it showed no inhibitory effect (or only a weak effect) on major commensal bacteria such as *E. faecalis *, *E. faecium *, *E. coli *, and *K. oxytoca *. Considering the fact that antimicrobial agents with spectra as broad as that of GSC have not been identified, GSC may be acting through a novel antimicrobial mechanism. Thus, our data support the empirical usage of GSC in intestinal infections.

Furthermore, GSC did not induce toxin production. Antimicrobial agents are known to induce the release of toxins from bacteria by disrupting their cell walls or by activating toxin-encoding phages [15, 16]. These data suggest that GSC inhibits intestinal pathogens without inducing toxin production.

This study has a major limitation. As this *Portulaca oleracea* L. formulation includes many components, the actual active component is still unknown [13]. Moreover, the activities of these compounds sometimes disappear during the isolation and extraction of the active components [17]. Future studies should thus focus on identifying the bioactive compounds in *Portulaca oleracea* L. formulations. However, our present data provide sufficient evidence to support the empirical use of GSC in infectious diseases.

Collectively, these findings indicate that GSC may be an effective treatment option for diarrhoea and a promising lead compound for the development of novel antimicrobial agents.
